# Pathogen Special: *Vibrio Cholerae, Pseudomonas Aeruginosa* and *Xylella Fastidiosa*

**DOI:** 10.1002/1097-0061(200012)17:4<307::AID-YEA51>3.0.CO;2-0

**Published:** 2000

**Authors:** Jo Wixon

**Affiliations:** School of Biological Sciences University of Manchester Stopford Building, Room 2.205, Oxford Road Manchester M13 9PT UK

## Abstract

One could almost say that it is the latest fashion to sequence a bacterial genome. However, this would belittle the efforts of those working on these important organisms, whose data will greatly help those working on the prevention of disease in the fields of medicine and agriculture. In this feature we present a guided tour of the latest additions to the ‘sequenced microbes’ club. *Vibrio cholerae* is the causative agent of cholera, which is still a threat in countries with poor sanitation and unsafe drinking water. *Pseudomonas aeruginosa* is responsible for a large proportion of opportunistic human infections, typically infecting those with compromised immune systems, particularly cystic fibrosis patients, those patients on respirators and burn victims. *Xylella fastidiosa* is a plant pathogen that attacks citrus fruits by blocking the xylem, resulting in juiceless fruits of no commercial value.


**Yeast**
*Yeast* 2000; **17**: 307–313.

**Featured Organism**

### Pathogen special: *Vibrio cholerae*, *Pseudomonas aeruginosa* and *Xylella fastidiosa*

Jo Wixon*
*School of Biological Sciences, University of Manchester, Stopford Building, Room 2.205, Oxford Road, Manchester M13 9PT, UK*

*Correspondence to: J. Wixon, School of Biological Sciences, University of Manchester, Stopford Building, Room 2.205, Oxford Road, Manchester M13 9PT, UK.*

Received: 4 October 2000
Accepted: 5 October 2000

### Abstract

**One could almost say that it is the latest fashion to sequence a bacterial genome. However, this would belittle the efforts of those working on these important organisms, whose data will greatly help those working on the prevention of disease in the fields of medicine and agriculture. In this feature we present a guided tour of the latest additions to the 'sequenced microbes' club. *Vibrio cholerae* is the causative agent of cholera, which is still a threat in countries with poor sanitation and unsafe drinking water. *Pseudomonas aeruginosa* is responsible for a large proportion of opportunistic human infections, typically infecting those with compromised immune systems, particularly cystic fibrosis patients, those patients on respirators and burn victims. *Xylella fastidiosa* is a plant pathogen that attacks citrus fruits by blocking the xylem, resulting in juiceless fruits of no commercial value. Copyright © 2000 John Wiley & Sons, Ltd.**

### *Vibrio cholerae*

### Background

*Vibrio* is a free-living, Gram-negative $\gamma$-proteobacterium that populates oceans, estuaries and coastal waters, and can be pathogenic to shellfish, finfish and mammals. *Vibrio cholerae* is shaped like a curved rod and has a single polar flagellum that it uses to move around when in its planktonic form. It can also exist as a non-motile biofilm, and under conditions of nutrient starvation it can enter a dormant, 'viable but non-culturable' state, which is thought to play a role in the emergence and persistence of pathogenic forms.

In humans, *V. cholerae* can colonize the surface of the small intestine, where it secretes a toxin, causing cholera. There have been seven cholera pandemics since 1871 and the disease has been epidemic in southern Asia for at least 1000 years (Wachsmuth *et al.*, [1994]). The toxin causes the secretion of water and electrolytes, resulting in watery diarrhoea. If untreated, onset is rapid and lethality can be high. Although our ability to treat the disease has improved over the last few decades, it is still a threat in countries where sanitation and the provision of clean drinking water are a problem. *V. cholerae* has been shown to be present in endemic regions during the periods between epidemics, inhabiting estuaries and brackish pools.

There are pathogenic and non-pathogenic species of *V. cholerae*, which vary in their virulence gene content. These genes include toxins, surface antigens and antibiotic resistance genes, the movement of which may be by phage-mediated lateral transfer, as pathogenicity islands, or orchestrated by other accessory elements.

### Genomics

The complete sequence of the *V. cholerae* El Tor N16961 genome was obtained by a whole genome shotgun approach by researchers at the Institute for Genome Research (TIGR, Heidelberg *et al.*, [2000]). The El Tor biotype is distinguished by the ability to produce haemolysins. There are two chromosomes; the largest is ca. 2.96 kb and the smaller chromosome is ca. 1.07 kb (Table 1). The larger chromosome (chromosome 1) has an origin of replication similar to those of *V. harveyi* and

Copyright © 2000 John Wiley & Sons, Ltd.

Table 1. Features of the *V. cholerae* genome

| Characteristic | Chromosome 1 | Chromosome 2 | Total |
|---|---|---|---|
| Size (bp) | 2 961 151 | 1 072 914 | 4 034 065 |
| G+C (%) | 47.7 | 46.9 | – |
| No. of ORFs | 2770 | 1115 | 3885 |
| No. similar to known proteins | 1614 | 465 | 2079 (53.5%) |
| No. similar to named protein of unknown function | 163 | 66 | 229 (6.0%) |
| No. of conserved hypotheticals | 478 | 165 | 643 (16.5%) |
| No. of hypotheticals (no homology) | 515 | 419 | 934 (24.0%) |

*Escherichia coli*, which show co-localization with dnaA, dnaN, recF and gyrA, like many prokaryote origins. In the case of the smaller chromosome (chromosome 2), the putative origin of replication has been assigned only on the basis of GC skew analysis and lacks recognition sites for the proteins that initiate replication. Chromosome 1 appears to be the native chromosome, carrying the genes for most essential growth and virulence functions, including a full set of rRNA and tRNA genes. The second chromosome contains a higher proportion of hypotheticals and genes with unknown function, but also several essential genes. It also contains an integron island (a gene capture system) and other features typically found on plasmids, indicating that it may have been a plasmid that was captured by an ancestral *Vibrio*. However, the GC percentages of the two chromosomes are very similar (Table 1), indicating that they have existed together for a long period. Thus it seems that the capture must be an early event in the evolution of the *Vibrio* genus. There is considerable evidence of interchromosomal recombination, with 105 duplicated genes having copies on both chromosomes. Regulatory interactions also exist between the two chromosomes, with regulatory genes expressed on one chromosome affecting the expression of genes on the other chromosome. *V. cholerae* will enable researchers to study chromosomal interactions and the replication and segregation of multiple chromosomes in a prokaryote system.

### Comparative genomics

The bulk of *V. cholerae* genes (1454) have highest homology to *E. coli* genes, the next closest relative being *Haemophilus influenzae*. A significant proportion (499 genes) are most similar to other *V. cholerae* genes. These are likely to be the products of recent duplications. Although many of these genes are of unknown function (Table 2), several of these genes have functions in chemotaxis and transport, indicating a role in foraging, which would imply that they aid the bacterium in surviving in a range of environments. There is clear evidence of lateral transfer events, with an example of a gene acquired from Archaea (3-hydroxy-3-methylglutaryl CoA reductase) and a gene found in two copies (glyA), one acquired from $\alpha$-Proteobacteria and one with $\gamma$-proteobacterial origin.

Table 2. Functional categories of *V. cholerae* duplicated genes

| Functional category | No. of ORFs |
|---|---|
| Hypothetical proteins | 113 |
| Conserved hypotheticals | 62 |
| Regulatory | 59 |
| Chemotaxis | 50 |
| Transport and binding | 42 |
| Transposition | 18 |
| Pathogenicity | 13 |

The El Tor N16961 strain (Heidelberg *et al.*, [2000]) has a single copy of the cholera toxin (CT) genes and other pathogenicity genes, including the RTX toxin genes on chromosome 1. The type IV pilus (TCP) genes are grouped in a proposed pathogenicity island also found on the larger chromosome. Trinucleotide composition analysis of the region in El Tor N16961 indicates that this is a region of atypical composition, which is flanked by bacteriophage-derived genes and has identical 20 bp sites at each end, these being the likely site of integration. The genes encoding *hap*, a haemagglutinin/protease that attacks proteins involved in tight junctions between epithelial cells (Wu *et al.*, [2000]), and *hlyA*, a haemolysin with enterotoxic activity (Alm *et al.*, [1988]), are located on chromosome 2. No shiga-like toxin genes were found, and there were no homologues of the

Copyright © 2000 John Wiley & Sons, Ltd.

*E. coli* heat stable toxin (ST), although these have been found in other *V. cholerae* strains (Ogawa *et al.*, [1990]).

### Future prospects

Expression studies and further comparisons between pathogenic strains and with non-pathogenic strains should aid our understanding of bacterial adaptation to varied environments. Global expression analysis has already been applied to the determination of genes whose expression changes following infection (Das *et al.*, [2000]). *V. cholerae* provides an interesting case of a multi-chromosome system in a prokaryote, and there is also the opportunity for further investigation to confirm the origin of the second chromosome.

### Websites

### General information

The Microtextbook – Cholera
**http://www.bact.wisc.edu/microtextbook/disease/cholera.html**

US FDA 'Bad Bug Book' – Cholera
**http://vm.cfsan.fda.gov/~MOW/chap7.html**

### Databases

The *Vibrio cholerae* Genome Database
**http://www.tigr.org/tdb/CMR/gvc/htmls/SplashPage.html**

NCBI *V. cholerae* page (has data on four strains of *V. cholerae*)
**http://www.ncbi.nlm.nih.gov/htbin-post/Taxonomy/wgetorg?name=Vibrio+cholerae**

### Pseudomonas aeruginosa

### Background

*Pseudomonas aeruginosa* is a rod-shaped Gram-negative bacterium. In its planktonic form, this is a fast-swimming bacterium that uses a single polar flagellum for movement. It can also exist as a sessile biofilm, attached by an alginate exopolysaccharide matrix to a wet surface. It can be found in moist environments such as soil and fresh or saltwater habitats.

*P. aeruginosa* is one of the most common causes of opportunistic infections in humans, and is able to infect a wide range of tissue types. It can also infect a range of animals and some plants. It is very rare for it to infect healthy individuals (it appears to be unable to achieve the first steps of pathogenesis) and usually affects those with compromised immune systems. It is the main cause of death in cystic fibrosis sufferers, whose lungs are particularly amenable to long-term infection. It is highly resistant to antibiotics and as a result, treatment often fails and around 80% of cases lead to death. The resistance is thought to have evolved as a result of exposure to a wide range of natural antibiotics (produced by competing bacteria) whilst in the soil, and other habitats, throughout its evolution. The lipopolysaccharide outer membrane of *P. aeruginosa* acts as a permeability barrier, affording protection against a wide range of antibiotics, and in the biofilm form it is even more resistant.

In keeping with pseudomonad family characteristics, it has a broad metabolic capacity, which allows it to grow on a wide range of energy sources. Having very few nutrient requirements, it is easily isolated from the environment and amenable to scientific study. It can even be found contaminating 'distilled water'.

### Genomics

*P. aeruginosa* has one large circular chromosome, ca. 6.3 Mb in size (Stover *et al.*, [2000]; Table 3), which is significantly larger than those of *Bacillus subtilis*, *Escherichia coli* and *Mycobacterium tuberculosis,* which are all between 4 and 5 Mb. The sequence of the genome of the PAO1 strain (Stover *et al.,* [2000]) contains almost as many genes as that of the yeast, *Saccharomyces cerevisiae* (which has 6200), implying greater complexity than many other bacteria. This strain, originally isolated from a wound (Holloway, [1955]), was chosen because it was the most commonly studied, with genetic and physical maps available to aid the construction of the sequence from the whole-genome-shotgun data.

The annotation of *P. aeruginosa* genes was a community project (PseudoCAP; see Websites), organized over the Internet (Brinkman *et al.*, [2000]). Volunteers were recruited, first across the *P. aeruginosa* community, then spreading out to

Copyright © 2000 John Wiley & Sons, Ltd.

Table 3. Features of the *P. aeruginosa* genome

| Characteristic | |
|---|---|
| Size (bp) | 6264403 |
| G + C (%) | 66.6 |
| No. of ORFs | 5570 |
| No. of ORFs of known (demonstrated) function | 372 (6.7%) |
| No. with high similarity to proteins of known function (in other organisms) | 1059 (19.0%) |
| No. with proposed function (motifs or lower similarity) | 1590 (28.5%) |
| No. of conserved hypotheticals | 769 (13.8%) |
| No. of hypotheticals (no homology) | 1780 (32.0%) |

researchers with interests in particular gene families and with experience of bacterial physiology. This resulted in the assignment of a functional class to 54.2% of the genes. Thirty-two per cent of the genes have no homologues amongst any of the reported sequences, and a further 13.8% have homologues with no known function (Table 3). G+C content and codon usage analyses revealed evidence of horizontal transfer events, identifying 10 regions over 3 kb in size that may have been acquired in this way, and also two probable bacteriophage integrations.

### Comparative genomics

In a comparison with 22 other bacterial genomes, *E. coli* appeared to be the closest relative to *P. aeruginosa*, accounting for just under half of the highest homologies to *P. aeruginosa* genes. A direct comparison between the two rules out large-scale duplication as the reason for the greater size of the *P. aeruginosa* genome. Local conservation of gene order is evident; 33 clusters of five or more ORFs with conserved order were found (allowing for single-ORF insertions or deletions), accounting for 256 ORFs. Only a few of these had been duplicated in either genome. Apart from the rDNA loci, the longest repeat in the *P. aeruginosa* genome is a duplicated gene cluster of only seven genes. A comparison with *B. subtilis*, *E. coli* and *M. tuberculosis* showed that *P. aeruginosa* has more distinct gene families, implying a wider range of functions – in fact it has half as many again as would be expected from a consideration of genome size.

Comparisons of the distribution of genes amongst functional categories showed that *P. aeruginosa* has the largest proportion of regulatory genes of all the sequenced bacterial genomes (between 8.4% and 9.4%, depending on annotation method). This is consistent with the trend (amongst prototrophic bacteria able to survive in diverse environments) that as genome size increases, so does the proportion of regulatory genes. The most striking result was obtained for the two-component regulator families, of which there were far more than in the other genomes analysed. These have roles in response to environmental changes, global regulation and regulation of virulence. There is also a larger number of outer membrane proteins than might be expected (ca. 150), including specific porins, Ton-B gated porins and OprM proteins involved in efflux or secretion. Almost 300 cytoplasmic membrane transport systems were identified (Genomic Comparisons of Membrane Transport Systems website). Whilst much of the substrate specificity profile was seen to match *E. coli* and *B. subtilis*, there were exceptions. *P. aeruginosa* seems deficient in sugar transporters, having no predicted major facilitator superfamily sugar transporters and only two phosphotransferase system sugar transporters. This correlates with its aerobic, oxidative metabolism (it lacks an intact glycolytic pathway). However, *P. aeruginosa* does have a wide range of $\beta$-oxidative metabolic genes, explaining its ability to grow on a wide range of alternative carbon sources. In addition, its complement of chemotaxis genes appears to be the most complex amongst the completed bacterial genomes, with four loci encoding chemotaxis signal transduction pathways.

### Future prospects

The genome has provided clues as to the high resistance of *P. aeruginosa* to antibiotics; a large number of drug efflux systems, in particular of the RND multidrug efflux system, were identified. For a review of these systems and new approaches that can be used to combat *P. aeruginosa,* see Hancock and Speert ( [2000]). There are also several findings that could explain the ability to survive in such a diverse range of environments. The chemotaxis pathways, the wide range of metabolic pathways and the broad transport capacity (both import and export) of *P. aeruginosa* allow it to cope with many challenges. All these functions require coordination, and this then could be the reason for the large number of regulatory genes, which will also be an interesting avenue for further investigation. The information generated from the sequence will play a

Copyright © 2000 John Wiley & Sons, Ltd.

crucial role in the search for new targets for antimicrobials aimed at treating *P. aeruginosa* infections.

### Websites

### General information

The Microtextbook – *Pseudomonas*
**http://www.bact.wisc.edu/microtextbook/disease/pseudomonas.html**

### Genome project

The *Pseudomonas* Genome Project
**http://www.pseudomonas.com**

*Pseudomonas Community Annotation Project (PseudoCAP)*
**http://www.cmdr.ubc.ca/bobh/PAAP.html**

University of Washington Genome Centre
**http://www.genome.washington.edu/UWGC/**

The Cystic Fibrosis Foundation
**http://www.cff.org/**

Pathogenesis Corporation
**http://www.pathogenesis.com/**

### Other sites

*Pseudomonas* Genetic Stock Centre
**http://core-admin.ecu.edu/Pseudomonas/**

Pre-completion Genome Database
**http://pseudomonas.bit.uq.edu.au/**

### Xylella fastidiosa

### Background

*Xylella fastidiosa* is a Gram-negative, rod-shaped, non-flagellate bacterium. It is highly specialized for survival in the foregut of the xylem-feeding homopteran sharpshooter leafhopper and in the xylem of plants. Once it has been injected inside the xylem of the plant during feeding by the leafhopper, it produces a xanthan-like gum and aggregates, blocking the flow of water and nutrients to the growing shoots. This causes the early production of small, hard, juiceless fruits and yellow spots (chlorosis) on the leaves. The current method for control of the disease is pruning infected trees and planting new orchards with uninfected stocks. Although initial infections are localized, the bacteria then spread throughout the tree over time. The earlier in the life of the plant that the infection occurs, the more severe are the symptoms; plants infected in the nursery rarely survive for more than 2 years.

The sequenced *X. fastidiosa* 9a5c clone (Simpson *et al.*, [2000]) was isolated from Valencia orange twigs affected by citrus variegated chlorosis (CVC). Other strains have been shown to affect other plants, including grapes, alfalfa, peaches, nuts and coffee. As its name implies, it is fastidious, having many nutritional requirements, which has made isolation and propagation for laboratory studies incredibly difficult. One of the advances that is hoped will come from the knowledge of the genome sequence is the ability to produce a medium for growth that matches its nutritional requirements.

### Genomics

The genome of *X. fastidiosa* is comprised of one ca. 2.7 Mb circular chromosome and two plasmids, ca. 51 kb and ca. 1 kb long, respectively (Table 4). The origin of replication on the chromosome has been identified by the presence of conserved motifs and a coincident inversion of the GC skew. There is also evidence of four sites of bacteriophage lambda integration. One prophage contains a pair of virulence-associated genes, a killer and a suppressor gene similar to those of an animal pathogen. Another contains an ORF similar to one encoding a component of the *Haemophilus influenzae* major pilus. In fact, as much as 7% of the genome may be phage derived, suggesting that bacteriophage-mediated horizontal gene transfer is a significant mechanism of gene content evolution in this bacterium. Four regions of the larger plasmid appear to be transposon-derived, suggesting yet another mechanism for lateral transfer of genes.

Of the 2848 predicted ORFs, only 47% could be assigned a putative function, a somewhat lower proportion than for *Thermotoga maritima* or *Neisseria meningitidis,* for example. The sequencing group suggest that this could, in part, be because this is the first plant pathogen to be sequenced.

Copyright © 2000 John Wiley & Sons, Ltd.

Table 4. Features of the *X. fastidiosa* genome

| Characteristic | Chromosome | Plasmid pXF51 | Plasmid pXF1.3 |
|---|---|---|---|
| Size (bp) | 2679305 | 51158 | 1285 |
| G + C (%) | 52.7 | 49.6 | 55.6 |
| No. of ORFs | 2782 | 64 | 2 |
| No. with functional assignment | 1283 | 30 | 1 |
| No. of conserved hypotheticals | 310 | 8 | – |
| No. of hypotheticals (no homology) | 1083 | 24 | – |

### Comparative genomics

Phylogenetic analysis of the 75 proteins from the 21 other sequenced bacterial genomes in the COG database (as of March 2000; Tatusov *et al.* [2000]) that showed homology to *X. fastidiosa* genes revealed that, in 69% of cases, *X. fastidiosa* was grouped with *H. influenzae* and *E. coli.*

*X. fastidiosa* has a similar complement of genes for transcriptional and translational functions and most of the genes for DNA repair mechanisms, when compared to *E. coli*, with the exception of the photolyase and the three polymerases induced by SOS. Despite being fastidious, it does have the full complement of genes for energy production and is able to use glucose, fructose, mannose and glycerol for glycolysis. The presence of cellulose degradation genes suggests that it can break down cellulose to help it cope with the low concentrations of monosaccharides in the xylem. There is no $\beta$-oxidation pathway and only limited catabolism of amino acids is available to the bacterium. The absence of a complete gluconeogenesis pathway suggests that it depends entirely on carbohydrates as a source of energy and anabolic precursors. It has a wide range of intact biosynthetic pathways, including most of the genes for the synthesis of all amino acids and fatty acids and complete pathways for the synthesis of nucleotides, enzyme co-factors and prosthetic groups. These are, it seems, essential requirements for a xylem dweller. In several cases, the missing enzymes are also missing in other Gram-negative bacteria, implying that these may be non-essential or that alternative genes exist that have not yet been identified by homology searches.

*Xylella* has 140 genes in the transport category, including representatives from several families. There are five receptors associated with iron transport, and in total 67 genes are involved in iron metabolism. The Brazilian team propose that it is the action of these transporters and also ones for uptake of other metal ions, such as manganese, that results in the variegated appearance of infected leaves by reducing the supply of these essential minerals.

Comparisons with *Xanthomonas campestris* have shown that the extracellular polysaccharide matrix, which allows *Xylella* to clump together, is synthesized by a subset of genes similar to the *X. campestris* genes that produce xanthan gum. It also appears that the regulation of this pathway is conserved between the two species (for a more detailed discussion of this comparison, see the review by Dow and Daniels, pp. 263–271).

The bacterium is thought to use fimbrial adhesion in its interactions with the insect host, the plant host and, indeed, itself. Twenty-six genes involved in the synthesis of Type 4 fimbria were identified. The group also found genes encoding homologues of afimbrial adhesins, previously only found in animal pathogens. Another previously animal pathogen-restricted gene set detected in *Xylella* is the haemagglutinins; three homologues of a *N. meningitidis* protein of this type were detected. Other virulence-related genes include two genes encoding potential pectolytic enzymes (although one has a frameshift), which would enable migration throughout the plant. *Xylella* also has five haemolysin-like genes, four of which belong to the RTX toxin family, and several other toxin genes. There are no avirulence genes (which define the host specificity of most phytopathogenic bacterium); this could be because they are not required due to the insect-mediated transmission of *Xylella* and its restriction to xylem, rather than infecting cells.

### Future prospects

The first hurdle for future studies of *Xylella* is to produce a medium that suits its nutritional requirements. Another useful tool for study is an amenable disease model. Work with tobacco has already

Copyright © 2000 John Wiley & Sons, Ltd. 

shown that it may well fulfil this role, showing typical disease symptoms ca. 2 months post-injection (Lopes *et al.*, [2000]). Potential functional studies include the establishment of which fimbria and afimbrial adhesins are responsible for adhesion to which host, and further studies into other aspects of its pathogenicity. These studies should provide clues as to new ways to treat citrus variegated chlorosis.

### Websites

### General information

*Xylella fastidiosa* website
**http://www.cnr.berkeley.edu/xylella/**

### Genomics

*Xylella fastidiosa* Genome Project
**http://onsona.lbi.ic.unicamp.br/xf/**

*Xylella fastidiosa* publications (Nature Genome Gateway)
**http://www.nature.com/genomics/papers/x_fastidiosa.htm**

*Xylella fastidiosa* functional genomics
**http://www.lbm.fcav.unesp.br/fun/**

*Xanthomonas citri* Genome Project
**http://watson.fapesp.br/xantho/main.htm**

### References

Alm RA, Stroeher UH, Manning PA. 1988. Extracellular proteins of *Vibrio cholerae* – nucleotide-sequence of the structural gene (hlyA) for the hemolysin of the hemolytic El-Tor strain 017 and characterization of the hlyA mutation in the non-hemolytic classical strain 569B. *Mol Microbiol* **2**: 481–488.

Brinkman FSL, Hancock REW, Stover CK. 2000. Sequencing solution: use volunteer annotators organized via Internet. *Nature* **406**: 933.

Das S, Chakrabortty A, Banerjee R, Roychoudhury S, Chaudhuri K. 2000. Comparison of global transcription responses allows identification of *Vibrio cholerae* genes differentially expressed following infection. *FEMS Microbiol Lett* **190**: 87–91.

Genomic Comparisons of Membrane Transport Systems: http://www-biology.ucsd.edu/~ipaulsen/transport

Hancock REW, Speert DP. 2000. Antibiotic resistance in *Pseudomonas aeruginosa*: mechanisms and impact on treatment. *Drug Resistance Updates* **3**: 247–255.

Heidelberg JF, Eisen JA, Nelson WC, *et al.* 2000. DNA sequence of both chromosomes of the cholera pathogen *Vibrio cholerae*. *Nature* **406**: 477–483.

Holloway BW. 1955. Genetic recombination in *Pseudomonas aeruginosa*. *J Gen Microbiol* **13**: 572–581.

Lopes SA, Ribeiro DM, Roberto PG, *et al.* 2000. *Nicotiana tabacum* as an experimental host for the study of plant–*Xylella fastidiosa* interactions. *Plant Dis* **84**: 827–830.

Ogawa A, Kato J, Watanabe H, Nair BG, Takeda T. 1990. Cloning and nucleotide-sequence of a heat-stable enterotoxin gene from *Vibrio cholerae* non-O1 isolated from a patient with traveler's diarrhea. *Infect Immun* **58**: 3325–3329.

Simpson AJG, Reinach FC, Arruda P, *et al.* 2000. The genome sequence of the plant pathogen *Xylella fastidiosa*. *Nature* **406**: 151–157.

Stover CK, Pham XQ, Erwin AL, *et al.* 2000. Complete genome sequence of *Pseudomonas aeruginosa* PAO1, an opportunistic pathogen. *Nature* **406**: 959–964.

Tatusov RL, Galperin MY, Natale DA, Koonin EV. 2000. The COG database: a tool for genome scale analysis of protein functions and evolution. *Nucleic Acids Res* **28**: 33–36.

Wachsmuth K, Olsvik Ø, Evins GM, Popovic T. 1994. In *Vibrio cholerae and Cholera: Molecular to Global Perspective*, Wachsmuth IK, Blake PA, Olsvik Ø (eds). ASM Press: Washington, DC; 357–370.

Wu ZY, Nybom P, Magnusson KE. 2000. Distinct effects of *Vibrio cholerae* haemagglutinin/protease on the structure and localization of the tight junction-associated proteins occludin and ZO-1. *Cell Microbiol* **2**: 11–17.

---

*Comparative Functional Genomics* is a cross-organism journal publishing studies on complex model organisms. This 'Featured Organism' article aims to present an overview of the chosen organisms, primarily for those working on other systems. It provides background information on each organism itself and a summary of the status of the study of the genome. It also gives a list of websites containing further information. These sections are a personal critical analysis of the current studies of the particular organism.

Copyright © 2000 John Wiley & Sons, Ltd.
